# Mapping Novel Pathways in Cardiovascular Disease Using eQTL Data: The Past, Present, and Future of Gene Expression Analysis

**DOI:** 10.3389/fgene.2012.00232

**Published:** 2013-05-31

**Authors:** Rajat M. Gupta, Kiran Musunuru

**Affiliations:** ^1^Department of Stem Cell and Regenerative Biology, Harvard UniversityCambridge, MA, USA; ^2^Division of Cardiovascular Medicine, Brigham and Women’s HospitalBoston, MA, USA

**Keywords:** cardiovascular disease, eQTL, GWAS, induced pluripotent stem cells, RNA-seq

## Abstract

Genome-wide association studies (GWAS) have identified genetic variants associated with numerous cardiovascular and metabolic diseases. Newly identified polymorphisms associated with myocardial infarction, dyslipidemia, hypertension, diabetes, and insulin resistance suggest novel mechanistic pathways that underlie these and other complex diseases. Working out the connections between the polymorphisms identified in GWAS and their biological mechanisms has been especially challenging given the number of non-coding variants identified thus far. In this review, we discuss the utility of expression quantitative trait locus (eQTL) databases in the study of non-coding variants with respect to cardiovascular and metabolic phenotypes. Recent successes in using eQTL data to link variants with functional candidate genes will be reviewed, and the shortcomings of this approach will be outlined. Finally, we discuss the emerging next generation of eQTL studies that take advantage of the ability to generate induced pluripotent stem cell lines from population cohorts.

Genome-wide association studies (GWAS) of common cardiovascular disease have successfully identified many new genetic loci that explain the heritability of myocardial infarction, dyslipidemia, hypertension, and diabetes (Hirschhorn, 2009). However, there is a substantial gap between single nucleotide polymorphism (SNP) associations discovered by GWAS and understanding how the locus contributes to disease. Only rarely does a SNP have a clear mechanistic relationship, such as when the polymorphism lies in the coding sequence of a gene and alters protein function, as has been observed with *NOD2* (nucleotide-binding oligomerization domain containing-2) mutations in inflammatory bowel disease (Hugot et al., 2001). A large number of GWAS hits lie in intergenic or intronic regions, where the link between variant and protein function is more circumspect.

Variation in gene expression is likely a major mechanism underlying susceptibility to complex diseases that is captured in many GWAS hits. A polymorphism in a regulatory element, for example, can alter the abundance of a gene transcript. The linkage disequilibrium based identification of loci for gene expression is termed expression quantitative trait locus (eQTL) mapping. Simply put, it is the combination of mRNA expression and genotype data to determine which variants correlate with transcript levels of genes. In principle, eQTLs can be mapped for known SNPs in any tissue type of interest. When the regulatory effect of a polymorphism is on a nearby gene the relationship is termed *cis*, whereas distant effects are termed *trans*. eQTL data can elucidate mechanisms underlying biological control of gene expression and the genes involved in disease pathogenesis.

Application of eQTL data to animal models and large human databases has established the heritability of gene expression (Dixon et al., 2007; Emilsson et al., 2008; Ding et al., 2010; Innocenti et al., 2011; Min et al., 2011). Furthermore, it has been shown that many common trait-associated variants act by altering transcript levels (Nicolae et al., 2010). Thus the use of eQTL data can provide the link between a SNP of interest and its responsible coding gene, and it is a reasonable initial line of inquiry in the search for functionally significant SNPs.

## eQTL Analyses and Blood Cholesterol Levels

A recent study used eQTL data to elucidate the relationship between a non-coding GWAS variant in a chromosome 1p13.3 locus associated with low-density lipoprotein (LDL) cholesterol and the hepatic expression of the *SORT1* gene (Musunuru et al., 2010). To determine if SNPs in the 1p13.3 locus were *cis*-acting regulators of nearby genes, expression levels were measured in liver, subcutaneous fat, and omental fat human tissue samples. After a large-scale analysis showed association between a representative SNP (rs646776) and transcript levels of three genes (*CELSR2*, *PSRC1*, and *SORT1*) in liver, a targeted eQTL analysis was performed in a replication cohort of 62 human liver samples. Minor allele homozygotes displayed more than 12-fold higher *SORT1* expression than major allele homozygotes. This differential gene expression by genotype was not apparent in the analysis of the adipose samples or in previously reported data from lymphocytes (Linsel-Nitschke et al., 2010), suggesting that the mechanism underlying the allele-specific gene expression is liver-specific. This finding paved the way for *in vivo* experiments modulating liver gene expression with siRNA or viral overexpression vectors, which established that *Sort1* alters blood LDL cholesterol levels by modulating hepatic lipoprotein particle secretion (Musunuru et al., 2010). The other two genes in the locus – CELSR2 and PSRC1 – were excluded as causal genes either through human eQTL data (CELSR2) or with similar *in vivo* viral overexpression in mice in mice (PSRC1; Musunuru et al., 2010).

With the successful use of eQTL data to link *SORT1* to cholesterol biology, the same approach is being utilized to uncover more genes of biological significance. Besides the *SORT1* locus, GWAS for blood cholesterol levels have also uncovered at least 94 other associated loci (Teslovich et al., 2010). To gain insight into how DNA variants in each of these loci might influence biologically relevant pathways, eQTL analysis was performed with RNA expression profiling of >39,000 transcripts in liver, omental fat, and subcutaneous fat. At a standard threshold of statistical significance (*P* < 5 × 10^−8^), 38 SNP-to-gene eQTLs in liver, 28 in omental fat, and 19 in subcutaneous fat were identified. Some lead SNPs were shown to be remote from their associated gene transcripts. For example, rs9987289 [associated with LDL-C and high-density lipoprotein (HDL)-C] correlated with a twofold change in liver expression of *PPP1R3B*, yet is 174 kb away from the gene. Similarly, rs2972146 correlated with *IRS1* expression in omental fat, despite being located 495 kb away from the gene. The eQTL data for these two loci provide another layer of insight into their biological significance.

These eQTL data were also used to nominate two additional genes for functional validation, the aforementioned *PPP1R3B*, and the *TTC39B* gene, each of which lies in or in proximity to a locus associated with HDL cholesterol (Teslovich et al., 2010). Although there are other genes at the loci in question, strong eQTL relationships between the lead SNPs and gene expression were only observed with *PPP1R3B* and *TTC39B*. Experiments in which the hepatic expression of each of the two genes was modulated in mouse liver established that they are both indeed regulators of blood HDL cholesterol levels and therefore are causal genes underlying the GWAS associations.

## eQTL Analyses to Uncover Novel Mechanisms in New Cell Types

One exciting prospect of GWAS is uncovering novel pathways and mechanistic insights into disease pathogenesis. With coronary artery disease (CAD), eQTL analyses in novel cell types are being undertaken to link genetic associations with pathways in inflammation and thrombosis. Monocytes represent one such cell type; its relative accessibility (from blood samples) and ease of use in eQTL studies make it an attractive cell type in which to link genotype and expression data, though it represents only one of the full spectrum of cell types that need to be studied to comprehensively find novel mechanisms for cardiovascular disease. One study linked two SNPs on chromosome 10q23.31 with increased CAD risk and increased *LIPA* mRNA expression in monocytes (Wild et al., 2011). The investigators began with a GWAS of 2,078 CAD cases vs. 2,953 control individuals and then went on to test all SNPs that reached genome-wide significance with monocyte transcripts in *cis* and *trans*. They found associations between *LIPA* mRNA transcript levels and low HDL cholesterol (*P* = 2.5 × 10^−3^) as well as impaired endothelial function measured by flow-mediated vasodilation, but not with traditional risk factors, LDL cholesterol, or triglycerides.

A second CAD-associated SNP did not show association with transcript expression in liver, whole blood, or monocytes, but instead was strongly associated with expression in aortic media, aortic adventitia, and mammary artery tissue (Folkersen et al., 2010). This SNP, located 117 kb downstream of the *PDGFD* gene (Coronary Artery Disease (C4D) Genetics Consortium, 2011), highlights the necessity of studying multiple cell types for expression data if the goal is to elucidate novel mechanistic pathways for complex diseases.

## Limitations of eQTL Analyses

For all the early success of eQTL analyses in uncovering new disease genes, the number of unresolved mechanistic links between SNPs and gene expression highlight the shortcomings of these data. Here we will discuss the technical and conceptual limitations of eQTL analysis, as well as the implications of and potential answers to these limitations.

### Identified eQTLs do not always lead to direct biological interpretation

Single nucleotide polymorphisms in the CAD-associated chromosome 9p21.3 locus – by far the strongest population-wide genetic contributor to CAD – have evaded mechanistic understanding though they are associated with not just CAD but also aneurysms, vascular disease, and multiple cancers (Helgadottir et al., 2008). From monocyte-derived eQTL data, there appear to be *cis* relationships with nearby genes *CDKN2A*, *CDKN2B*, and *ANRIL* (Cunnington et al., 2010). But these data do little to elucidate the mechanism by which the 9p21 locus contributes to disease. For a biologic connection between a SNP and a mechanism of disease much more experimentation is required; though eQTL analysis may provide the initial insight.

### Technical limitations: Platform coverage, batch variation, sample size, and tissue availability

There currently exist several commercial platforms by which expression data is obtained. The coverage of each platform is variable, and each is subject to its individual errors. This variability results in batch-to-batch variation between analyses that should otherwise be consistent. This was observed in a report of large-scale differences in gene expression between ethnic groups (Akey et al., 2007). In this case, the highly significant differences were found to be due to the separate processing of lymphoblastoid cell lines (LCLs) from subjects of European vs. Asian ancestry. Different microarray platforms only have 30–40% overlap in transcript detection (Barnes et al., 2005; Pedotti et al., 2008). With the advent of direct ultra-high-throughput sequencing of RNA transcripts (RNA-seq), analyses of gene expression are expected to improve; however, handling the terabytes of data generated by such approaches will be a challenge in its own right.

Suggested solutions for batch-to-batch variation include experimental and statistical approaches. Efforts should be made to spread sample groups across different processing times to limit this as a confounding variable. To correct for batch effects with statistical analyses, the possible variable that could contribute should be identified and reported. Published results, for example, should report the processing group and time of samples in a study (Leek et al., 2010).

Many eQTL databases are limited to a few 100 human samples because of cost and difficulty in obtaining tissue. This results in nominal *P* values in most eQTL analyses that are difficult to interpret (Schadt et al., 2003; Morley et al., 2004). To rectify this limitation, the National Institutes of Health have launched a 2-year pilot project to build a more comprehensive bank of samples termed the Genotype-Tissue Expression project (GTEx). To date, no project has analyzed genetic variation and expression in as many tissues in such a large cohort as planned for GTEx. If completed, GTEx will house samples of 30 different tissues from 1,000 donors. Even with this large databank, however, questions remain such as whether healthy or diseased samples should be collected together (Cookson et al., 2009).

The fact that many eQTL studies are conducted in LCLs is both a technical and conceptual shortcoming. LCLs form the basis for numerous gene expression studies because they represent an easily banked source of nucleic acids for genetic studies (Cookson et al., 2009). LCLs, however, exhibit genomic instability with multiple passages of storage and regrowth. Furthermore, LCLs likely have unique, tissue-specific expression profiles that may not reflect normal human biology. The predominance of clonal cell populations, for example, can result in random patterns of monoallelic expression (Plagnol et al., 2008). There is recent evidence, however, that other cell types have expression overlap with LCLs. In fact, 70% of *cis*-eQTLs in LCLs are shared with skin cells (Ding et al., 2010). This finding can allow for comparisons between tissue types, and conclusions about the functional importance of variants can potentially be made on this basis.

Even when eQTL studies use cells other than LCLs, there are inherent biases as a result of the limitations of tissue ascertainment. Many of the studies that profile liver and adipose expression use human samples obtained during surgeries (Schadt et al., 2008); it stands to reason that these were from non-healthy patients undergoing surgery for non-research-related reasons, since it would be unethical to perform such biopsies in healthy individuals. Thus the samples do not represent the general population, nor could they be ascertained *a priori* on the basis of particular genotypes or phenotypes of interest. Furthermore, the samples were limited in size, non-renewable, and could not be used to generate large numbers of cultured hepatocytes and adipocytes, since unlike LCLs primary hepatocytes and adipocytes cannot be sustained for long in tissue culture conditions.

## Future Directions

The full spectrum of gene expression that relates to disease involves not only many cell types but different conditions for these cells to “exercise the genome.” There is evidence that environmental actions on gene expression are profound in humans, and, where possible, future eQTL studies should incorporate environmental stimuli. Model stimuli that could be tested in an *in vitro* system include pro-inflammatory stresses, metabolic stresses (such as hypoglycemia or hypoxia), the response to radiation, and even response to drugs, hormones, and peptides (Cookson et al., 2009).

The next generation of eQTL studies will have the ability to utilize induced pluripotent stem cells (iPSCs) as a renewable source of patient-specific cell lines. Recent advances in nuclear reprogramming technology allow for the transformation of terminally differentiated adult cells into induced iPSCs that are phenotypically indistinguishable from embryonic stem cells (Takahashi et al., 2007). This leap forward makes possible the creation of patient-specific iPSC lines that, by definition, can be maintained in culture indefinitely. Gene expression profiling from these patient-specific cell lines will allow larger-scale gene analyses from patients in whom complete phenotype data is available.

As one example, an effort is now underway to create iPSCs from blood samples from up to 3,000 participants in the Framingham Heart Study (FHS), which is a unique, community-based cohort in which three generations of individuals have been extensively phenotyped and genotyped. Although limited eQTL analyses have been performed within the FHS (Levy et al., 2009; Fox et al., 2011), these analyses suffer from the standard pitfalls of underpowered results and suboptimal tissue types. With iPSCs, the full spectrum of gene expression can be extensively profiled in any tissue that can be differentiated from the cell lines, allowing investigators to study tissue types that in ordinary circumstances would be prohibitive to obtain from living human beings. In the planned pilot studies, a subset of the FHS iPSC lines will be differentiated into hepatocytes and adipocytes – two tissues of relevance to cardiovascular and metabolic diseases – followed by whole-genome gene expression profiling, which will pave the way for eQTL analyses of unprecedented size and rigor.

## Conclusion

Gene expression analyses can yield important information about genetic architecture and can point to mechanisms that link genetics and cardiovascular disease. The application of eQTL analyses has already forged connections between GWAS data and mechanistic pathways. Technical and conceptual limitations, however, limit the ability to systemize this approach to more polymorphisms for more diseases. The advent of more comprehensive biobanks, high-throughput RNA sequencing, and collections of iPSC lines holds much promise for the field. Ultimately, the ability to explain the heritability of common diseases such as cardiovascular and metabolic disorders should be greatly facilitated by future eQTL efforts.

## Conflict of Interest Statement

The authors declare that the research was conducted in the absence of any commercial or financial relationships that could be construed as a potential conflict of interest.
